# Population‐Based Study Found Low Risk of Misdiagnosing Long QT Syndrome as Breath‐Holding Spells in Swedish Children

**DOI:** 10.1111/apa.70460

**Published:** 2026-01-22

**Authors:** Sanna Hellström Schmidt, Ida Jeremiasen, Erik A. Eklund, Cornelis Jan Pronk

**Affiliations:** ^1^ Department of Pediatrics, Clinical Sciences Lund Lund University Lund Sweden; ^2^ Childhood Cancer Center Skåne University Hospital Lund Sweden; ^3^ The Pediatric Heart Center Skåne University Hospital Lund Sweden; ^4^ Department of Pediatric Neurology Skåne University Hospital Lund Sweden; ^5^ Wallenberg Centre Molecular Medicine and Division Molecular Hematology Lund University Lund Sweden

**Keywords:** breath‐holding spells, electrocardiography, long QT syndrome, misdiagnosis

## Abstract

**Aim:**

An electrocardiogram is commonly recommended in breath‐holding spell management, mainly to rule out long QT syndrome. This retrospective study investigated the risk of long QT syndrome being misdiagnosed as breath‐holding spells in a paediatric population in southern Sweden.

**Methods:**

Data on patient characteristics and diagnostic findings were reviewed for patients aged < 10 years who were diagnosed with long QT syndrome between 2004 and 2018.

**Results:**

Sixteen children were diagnosed with long QT syndrome; 10 were diagnosed through genetic screening, 4 following perinatal cardiac symptoms, and 2 due to episodes of syncope or seizures later diagnosed as epilepsy and breath‐holding spells. Three patients were > 24 months old at suspicion of long QT syndrome, and 10 were < 3 months old. No patient exhibited symptoms directly attributable to long QT syndrome, and no diagnosis of long QT syndrome was delayed due to suspicion of or misdiagnosis as breath‐holding spells.

**Conclusions:**

The number of symptomatic long QT syndrome cases overlapping with the presentation of breath‐holding spells is likely small. The findings of this study suggest that children < 3 months old with suspected breath‐holding spells should undergo an electrocardiogram.

## Summary

- Electrocardiogram is routinely advised in breath‐holding spell management to exclude long QT syndrome.
- We found no cases of misdiagnosis between breath‐holding spells and long QT syndrome in a paediatric population of 16 children with long QT syndrome in southern Sweden.
- An electrocardiogram might not be warranted in all cases of suspected breath‐holding spells, but some caution may be needed in children < 3 months old.

**Abbreviations**
BHS: breath‐holding spells
ECG: electrocardiogram
ICD‐10: International Classification of Diseases, Tenth Revision
LQTS: long QT syndrome
QTc interval: corrected QT interval

## 1 Introduction

Long QT syndrome (LQTS) encompasses a group of cardiac channelopathies affecting approximately 1 in 2000 live births [1]. LQTS is typically inherited in an autosomal dominant manner, but sporadic cases with *de novo* pathogenic variants exist. Symptoms related to a prolonged corrected QT (QTc) interval include syncope, aborted cardiac arrest, and potentially fatal ventricular arrhythmias [1, 2, 3, 4, 5, 6, 7]. Triggers of arrhythmia and the clinical course vary among the different genetic subtypes, but the majority of LQTS cases belong to types LQT1–3, corresponding to variants in *KCNQ1*, *KCNH2* and *SCN5A* [2, 3, 4, 8]. As LQTS is a monogenic condition, genetic testing has become a diagnostic mainstay [1, 8].

Breath‐holding spells (BHS) are non‐epileptic events [9, 10] with a reported incidence of 4.6%–27% in paediatric cohorts [9, 11]. The spells follow a typical sequence of events initiated by a trigger, most commonly an emotion, followed by apnea and a change in facial colour to cyanotic or pallid. In severe spells, loss of consciousness and motor seizures may occur [9, 10, 11]. Spells typically first occur before 24 months of age and abate before school age [9, 10, 11, 12, 13, 14, 15]. Several studies have reported a family history of BHS [10, 11, 12, 13, 15, 16, 17, 18]. Anamnesis is the most important tool for diagnosing BHS.

The published literature on BHS commonly recommends performing an electrocardiogram (ECG) during the initial management of suspected BHS. This is primarily because of the risk of confusing symptoms of LQTS with those of BHS. This recommendation appears to be based on a few case reports of such misdiagnoses and the assumption that the clinical presentations of both conditions can be similar [19, 20, 21]. Studies on this topic are scarce because they are challenging to conduct, partly due to the low prevalence of LQTS [1]. Thus, evidence that an ECG should be performed in all children with BHS is insufficient [22, 23, 24]. In addition, Robinson et al. [23] found no overrepresentation of BHS in a cohort of children with LQTS, suggesting that the two diseases do not share a common pathology.

The exact mechanism for the occasional loss of consciousness in BHS is not fully understood but may involve autonomic nervous system immaturity or pathology [16, 25]. In LQTS, cardiac syncope due to hypoxia secondary to ventricular arrhythmias can lead to convulsions, sometimes referred to as torsadogenic seizures [20, 26]. LQT1 has the greatest overlap in age at symptom onset with BHS, though the mean and median ages are still higher in LQT1 [4, 5, 6, 7, 27, 28]. Unlike BHS, LQT1 symptoms are often triggered by exercise. In contrast, LQT2 symptoms are reported to be triggered by loud noises, arousal, and emotional stress, which indicates some overlap with triggers of BHS [2, 28]. However, LQT2 symptoms typically present in adolescence, an age when BHS have normally disappeared [2, 28]. MacCormick et al. reported that 40% of patients with LQTS or Brugada syndrome experienced a change in colour to blue or grey during syncope. This is also commonly seen in BHS. However, the duration of unresponsiveness was longer in these patients than in typical BHS [3, 22].

We previously reported, in a cohort of 519 children diagnosed with BHS in southern Sweden between 2004 and 2018, that ECGs were performed in 45.1% of patients. None revealed a prolonged QTc interval or other pathologies that could explain the BHS symptoms [22]. Based on these findings, we proposed guidelines for the management of patients with typical BHS. These guidelines were expected to reduce the number of ECGs in BHS management. The recommendation for ECG was limited to children older than 24 months at the presentation of severe spells [22]. The guidelines focused on anamnesis and physical examination, with prompts for questions on the heredity of cardiac disease or sudden death and situations suspicious of a cardiac cause (e.g., events in water or nocturnal events in a relative or the patient). An ECG was also recommended in patients with a known heart condition, chest pain, heart murmur or QT‐prolonging medications, as well as a previous pathological ECG.

This study aimed to assess the risk of misdiagnosing LQTS as BHS by investigating the diagnostic methods, patient characteristics, and symptoms in a cohort of children diagnosed with LQTS.

## 2 Patients and Methods

### 2.1 Study Design and Population

This retrospective cross‐sectional study included patients from the same southern Swedish population and spanned the same period as the study focusing on BHS by Hellström Schmidt et al. [22]. All children < 10 years old residing in the Skåne Region in southern Sweden who received the International Classification of Diseases, Tenth Revision (ICD‐10) diagnosis *I45.8 Long QT syndrome* between 1 January 2004 and 31 December 2018 were included in this study. The patients were identified using the Patient Administrative Support Registry.

In 2021, data on patient characteristics, symptomatology, ECG parameters, comorbidities, family history and results of other diagnostic interventions were manually extracted from digital medical records in Melior software (Siemens AB, Munich, Germany) according to a REDCap data entry form (Table S1). The investigators did not review the LQTS diagnosis retrospectively, but genetic testing results were documented. The symptoms reported in this study were described by patients and caregivers.

The diagnosis date was the date the ICD‐10 diagnosis was registered. The date of initial LQTS suspicion was the first mention of LQTS in the medical records regardless of subsequent dismissal. All comorbidities were registered, including premature birth. Manual ECG interpretations by physicians were prioritized over machine interpretations when both were available. The investigators did not retrospectively interpret patient ECGs. Normal, borderline, and pathological QTc intervals were based on clinical physician assessments if noted in the patient records. These were compared with Swedish national guidelines for LQTS [29]. The QTc interval for children aged 1–15 years was classified according to these guidelines: < 440 ms, normal; 440–460 ms, borderline; > 460 ms, pathological. The guidelines had a 480‐ms threshold for neonates.

### 2.2 Statistical Analysis

Descriptive statistics were calculated using data from our REDCap (version 10.0.33) [30] database and analysed in Excel (Microsoft Corp, Washington, US). Continuous variables are reported as medians (with ranges) due to the small sample size. When applicable, categorical variables are presented as frequencies and percentages. Missing data are indicated as unknown.

### 2.3 Ethics

Approval from the Swedish Ethical Review Authority was obtained (Dnr 2019–00374 and amendment application, Dnr 2020–06944). Authorization for health‐care data collection and storage was granted by the Skåne Region (KVB nr 027–19). All research was performed in accordance with relevant regulations and guidelines.

## 3 Results

### 3.1 Patient Characteristics

The study included 16 patients (10 females, 6 males) diagnosed with LQTS. Five patients were index cases; in the remaining 11 cases, heredity was known or suspected at the time of initial suspicion of LQTS in the child. Three pairs of siblings (patients 4 and 10, 12 and 14, 15 and 16) and one pair of more distant relatives (patients 1 and 2) were present in the cohort.

The median age at diagnosis was 17.5 (range, 0–118) months in the whole cohort and 18 (0–55) months among index cases (Table 1). Five patients, including one index case, were < 3 months old at diagnosis. Seven patients, including two index cases, were older than 24 months at diagnosis.

**TABLE 1:** Diagnostic pathway from suspicion of LQTS to diagnosis.

Patient	Age at initial suspicion (months)	Age at diagnosis (months)	Comment on the time from suspicion to diagnosis	Mode of diagnosis	QTc interval (ms) at diagnosis^a^	Type of LQTS
*Index cases*						
1	0	18	Diagnosis discussed and decided at arrhythmia conference more than 1 year before the official ICD‐10 diagnosis	ECG for other reason: bradycardia and arrythmia (SVT, AV‐block, bradycardia, and TdP) after birth	650	LQT2
2	0	0	Heredity not known, prolonged QTc interval at birth	ECG for other reason: prenatally diagnosed bradycardia plus a heart murmur and early second tone on auscultation	540	LQT1 (homozygote)
3	1	55	Prolonged QTc interval noted after birth, patient followed by cardiologist until genetic testing	ECG for other reason: heart murmur after birth	445	LQT1
4	11	17	Diagnosis before genetic testing because of parent with LQTS (found after suspicion was raised in the patient but parent received genetic testing result before the patient did) and long QTc interval on ECG	ECG for other reason: extremely premature birth together with persistent ductus arteriosus and mild pulmonary valve stenosis	467	LQT2
5	26	42	LQTS diagnosis falsely dismissed, 16‐month diagnostic delay	ECG for other reason: seizures (epilepsy)	458	LQT1
*Cases with suspected or known heredity*						
6	0	0	Diagnosed in Iceland perinatally	Screening because of heredity	Unknown	LQT1
7	0	37	Diagnostic delay waiting for genetic testing in patient	Screening because of heredity	472	LQT1
8	0	0	Heredity and neonatal ECG with prolonged QTc interval manually calculated	Screening because of heredity	473	LQT2
9	0	14	Almost 1 year from the genetic testing result to the date of the official diagnosis	Screening because of heredity	400	LQT2
10	0	0	Known heredity	Screening because of heredity	418	LQT1
11	0	2	Known heredity	Screening because of heredity	500	LQT2
12	1	118	ECG normal at birth, long diagnostic delay waiting for genetic testing of family members	Screening because of heredity	460	LQT2
13	14	16	Episodes suspected to be LQTS‐related because of known heredity, no diagnostic delay	Suspicion of LQTS symptoms: syncope and seizures (breath‐holding spells)	470	LQT1
14	21	93	Sparse contacts with cardiologist from young age until diagnosis (normal QTc interval manually calculated)	Screening because of heredity	415	LQT7 (KCNJ2)
15	29	91	Long diagnostic delay waiting for parental genetic testing	Screening because of heredity	445	LQT1
16	43	45	Tested after other relatives were diagnosed with LQTS	Screening because of heredity	479	LQT1

Abbreviations: AV, atrioventricular; ECG, electrocardiogram; LQTS, long QT syndrome; QTc interval, corrected QT interval; SVT, supraventricular tachycardia; TdP, torsades de pointe.

^a^ From the ECG closest in time to diagnosis.

Median age at suspicion of LQTS was 0.5 (range, 0–43) months in the whole cohort and 1 (range, 0–26) month among index cases. Ten of the 16 patients, including 3 index cases, were < 3 months old at suspicion of LQTS. Three patients, including one index case, were older than 24 months at suspicion of LQTS. Suspicion preceded diagnosis with a median of 10 (range, 0–117) months.

### 3.2 Diagnostics

The genetic type had been resolved in all 16 patients: LQT1 in 9 (56.3%), LQT2 in 6 (37.5%), and LQT7/Andersen‐Tawil syndrome in 1 (6.3%). Among the index cases, three had LQT1 and two had LQT2. A family history of LQTS was found in 11 patients at diagnosis and in another 4 patients after diagnosis and family screening. One patient had a *de novo* pathogenic variant.

**FIGURE 1:**
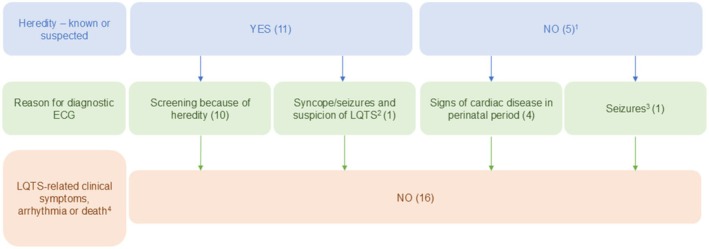
Schematics of heredity, indication for the diagnostic electrocardiogram (ECG), and adverse events. The number of patients is given in parentheses. ^1^The five patients included one case with a *de novo* pathogenic variant and four cases of unknown heredity. ^2^The patient was diagnosed with long QT syndrome (LQTS) and suspected breath‐holding spells. ^3^The patient was diagnosed with LQTS and epilepsy. ^4^The data were obtained from medical files.

Ten patients (62.5%) were diagnosed following screening due to a relative's suspected or confirmed LQTS diagnosis (Figure 1). Four patients (25.0%) initially not suspected of having LQTS were diagnosed after ECGs were performed in conjunction with signs of potential cardiac disease during the perinatal period (Table 1). Two patients (12.5%) experienced syncope and/or seizures that contributed to the diagnosis of LQTS. One of these patients had suspected febrile seizures at 25 months of age. ECGs showed QTc intervals of 460–480 ms. After 2 months, the suspicion of LQTS was dismissed upon normal telemetry monitoring during a seizure. Suspicion was renewed after the patient presented with different kinds of seizure semiology. The other patient had two episodes of suspected LQTS‐related syncope at around 14 months of age. The QTc interval was 464 ms. LQTS was diagnosed after genetic testing, probably prompted by the recent diagnosis of LQTS in a first‐degree relative. The syncope episodes continued and were extensively investigated despite the suspicion of BHS due to some concerning features during spells. At 33 months of age, the spells were deemed to be BHS. Both patients had an internal loop recorder implanted for ECG recordings and to differentiate between a seizure disorder and LQTS. No LQTS‐related arrhythmias were recorded during follow up in these patients, which lasted for 34 and 39 months, respectively.

**FIGURE 2:**
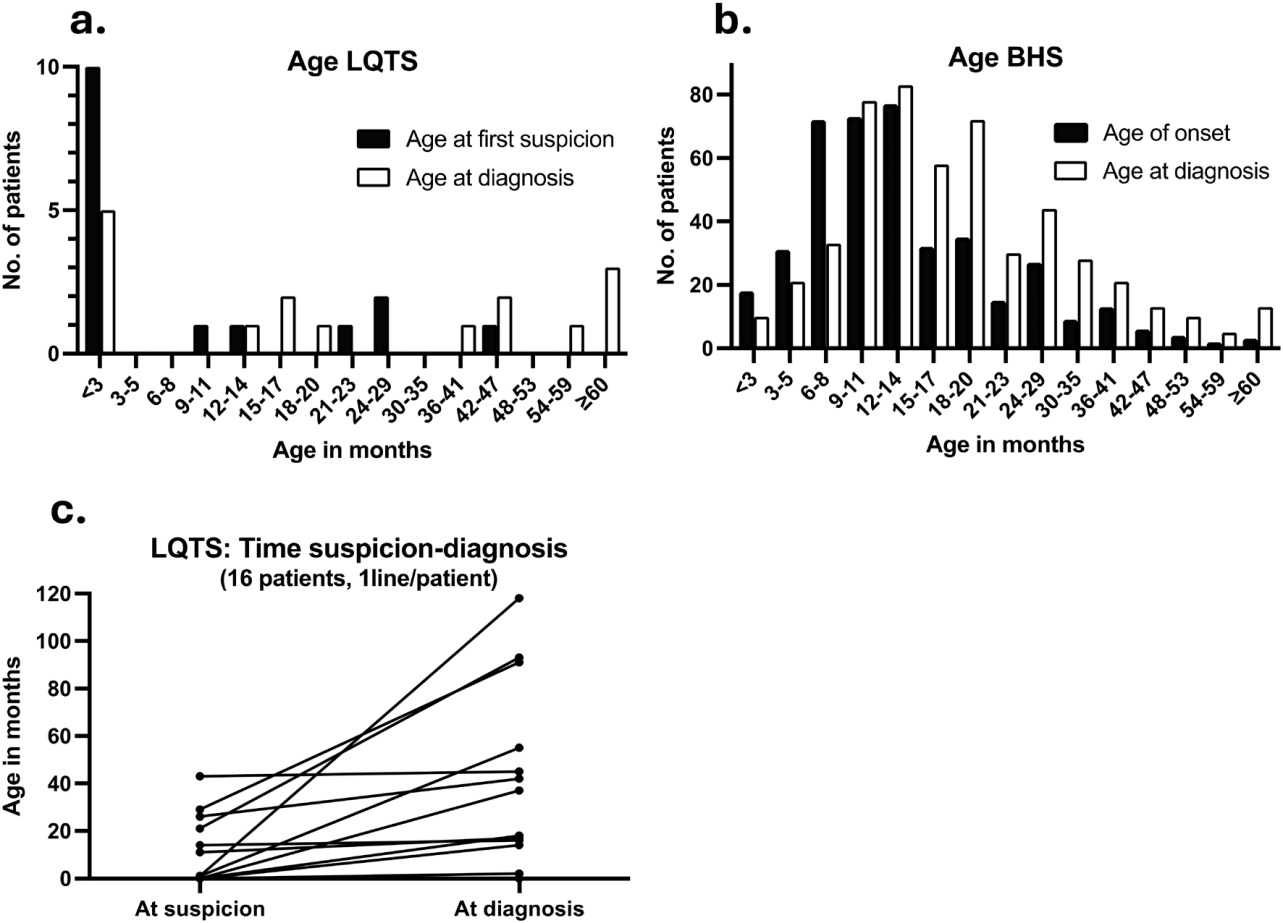
Age at suspicion and diagnosis of long QT syndrome (LQTS) and breath‐holding spells (BHS). (a, b) Total number of patients in a given age interval at suspicion and diagnosis of LQTS (a) and age of onset and diagnosis of BHS (b). (c) Time between suspicion and diagnosis of LQTS. A total of 16 patients were included, with each line representing one patient.

The time from the initial suspicion of LQTS to the registered diagnosis varied greatly (Figure 2c and Table 1). We observed some overlap in age at suspicion and diagnosis of LQTS (Figure 2a,b) compared with the cohort of 519 BHS cases described in our previous report [22]. However, in most patients, the age at suspicion of LQTS were before 3 months (Figure 2a), whereas BHS more commonly presented at an older age (Figure 2b).

The median QTc interval at diagnosis was 467 (range, 400–650) ms. Three patients had normal QTc intervals, five had borderline values, and seven had pathological values based on the national guidelines for LQTS in Sweden [29]. Among the index cases, the median QTc interval at diagnosis was 467 (range, 445–650) ms, with two cases considered borderline and three considered pathological. However, interpretations of the QTc interval by physicians during follow up varied significantly within each patient.

In 14 patients (87.5%), an ECG was conducted prior to the diagnostic ECG (Table 1). In five of the patients, this first ECG was not performed due to suspected LQTS; four ECGs were linked to signs of cardiac disease at birth and one following seizures. LQTS was mistakenly dismissed in the case following seizures. In eight patients, the indication for the initial ECG was LQTS screening. In one patient, LQTS was suspected because of syncope and a family history of LQTS, but the loss of consciousness was later attributed to BHS. Three patients (21.4%) with a positive family history of LQTS initially had a normal screening ECG.

### 3.3 Symptomatology

Nine patients (56.3%) reported symptoms prior to, concurrent with, or after the LQTS diagnosis (Table 2). However, interestingly, no symptoms were definitively associated with LQTS and the patients did not present with symptoms associated with BHS. An exception was the patient diagnosed with both LQTS and BHS described above.

**TABLE 2:** Symptoms reported by patients and parents.

Patient	Symptoms before, at, or after diagnosis of LQTS	Type of reported symptoms	Assessment of symptoms in medical files
*Index cases*			
1	None		
2	None		
3	Before	Palpitations	Assessed as most likely not related to cardiac disease
4	After	Four short absence attacks	Interictal EEG and 24‐h ECG were normal
5	Before, at, and after	Various types of seizures, including generalised tonic–clonic seizures (lasting 1 min to several minutes), absence episodes (lasting a few seconds to 5 min) and spells involving loss of tone and bradycardia (lasting several minutes to > 10 min)	Assessed after initial dismissal of the LQTS diagnosis as epilepsy‐related; no LQTS‐related arrhythmias registered on loop recording device
*Cases with suspected or known heredity*			
6	After	Suspected febrile seizure; one bout of syncope and one of vertigo (in sitting position)	Assessed as most likely not related to cardiac disease
7	After	Chest pain; vertigo and loss of consciousness on one occasion	Assessed as most likely not related to cardiac disease
8	None		
9	None		
10	None		
11	None		
12	Before, after	Before: tired, blue lips (gone at diagnosis), suspected febrile seizures After: tired	Possibly febrile seizures; assessed as most likely not related to cardiac disease
13	Before, at, and after	Episodes were triggered by falling on her butt and anger, followed by a deep breath or screaming; she became blue around the lips, ‘lifeless’, and limp for less than a second	Initially suspected to be LQTS‐related; later assessed as BHS; no LQTS‐related arrhythmias registered on loop recording device
14	After	Palpitations	Possibly LQTS (symptoms during period of low compliance with medication)
15	None		
16	After	Tiredness, headache, vertigo	Assessed as most likely not related to cardiac disease

Abbreviations: BHS, breath‐holding spells; ECG, electrocardiogram; EEG, electroencephalogram; LQTS, long QT syndrome.

No LQTS‐related cardiac events were documented during follow up, and none of the patients died. The median follow up was 79 (range, 9–147) months.

All patients were treated with beta‐blockers and one patient who experienced arrhythmias after birth was fitted with a pacemaker. No patient received an implantable cardioverter‐defibrillator during the study period.

## 4 Discussion

This study included 16 children diagnosed with LQTS during a 15‐year period in southern Sweden. During the same time period and geographic area, 519 patients were diagnosed with BHS [22]. In that previous cohort, 45.1% had an ECG performed and none were diagnosed with LQTS. In addition, none of the 16 patients diagnosed with LQTS had their diagnosis delayed due to a misdiagnosis of BHS. However, the patient diagnosed with epilepsy in whom the LQTS diagnosis was initially dismissed shows the diagnostic challenge when a physician encounters a small child with unclear episodes of syncope. In such patients, a family history of cardiac disease should lead to a referral for an ECG. Notably, the algorithm for the management of BHS we previously proposed [22] suggests ECGs for all BHS patients with a positive family history of cardiac disease or sudden death and when onset of severe BHS occurs after 24 months of age.

The onset of spells is the earliest opportunity for diagnosing BHS, whereas the initial suspicion date is the closest approximation for asymptomatic LQTS patients. Thus, the mean age at initial suspicion of LQTS can be used in comparisons with the age at onset in BHS. We found that 43.8% (*n* = 7) of patients in this LQTS cohort were older than 24 months at diagnosis, whereas only 18.8% (*n* = 3) were older than 24 months at initial suspicion of LQTS. In the previous BHS cohort, 74.2% of patients were diagnosed before the age of 24 months and 84.7% experienced the onset of spells before this age [22]. Despite the higher median age at diagnosis of LQTS compared with BHS [9, 11, 12, 13, 14, 15, 22], the ages overlap. The age of initial suspicion of LQTS in the present cohort placed all patients within the possible age range for BHS and the majority within the typical age range for onset of spells. Notably, the suspicion of LQTS was raised before 3 months of age in 62.5% of LQTS patients in the present cohort. In comparison, only 6.2% of BHS patients in the previous cohort experienced an onset of spells by 3 months of age.

In three of the five index cases, suspicion of LQTS was raised before 3 months of age and the median age at suspicion was 1 month. Previous studies observed early LQTS diagnosis together with indications that this patient group may be at risk of a more severe clinical disease course [4, 5, 7]. A large proportion of LQTS patients being diagnosed before the age of 3 months may challenge our recommendations to perform ECG only in patients older than 24 months unless a family history of cardiac disease or sudden death is present [22]. Early diagnosis is often made in asymptomatic patients before the age of 3 months through screening due to a positive family history of LQTS. All neonatal patients with LQTS in this study were asymptomatic. An expansion of the age groups in which an ECG is recommended would add few ECGs to the suspected BHS patient group in total. However, it would also direct the recommended ECGs towards an age group in which BHS is less common, cardiac symptoms are possibly harder to detect, and a greater need to consider differential diagnoses exists.

Given that patients with known or suspected heredity for LQTS would be identified by the questions prompted in our management algorithm for BHS, index cases were of primary interest. As most patients in our cohort were diagnosed through hereditary screening, only five patients were identified as index cases. None of the patients in this cohort presented with reported symptoms of LQTS [3, 4, 5, 6, 7], such as syncope or aborted cardiac arrest, without an alternative diagnosis that explains the symptoms. As such, a comparison with potential overlapping symptoms in BHS was difficult. The REDCap data entry form in Table S1 shows all variables we intended to capture, but only positive results are reported in the Results section, limiting the comparison. MacCormick et al.'s study with a comparable design and objectives identified a higher proportion of symptomatic LQTS patients [3]. In addition, unlike the current study, their cohort underwent genetic screening in an earlier era (2000–2005) and included a broader age range, with only one LQTS patient falling within the age range for BHS onset. Therefore, these cohorts are not fully comparable. An inclusion age < 10 years, greater awareness of LQTS, and/or more active genetic screening in patients with a positive family history may explain why most of the patients in our cohort were diagnosed pre‐symptomatically. Previous research indicates that the risk of cardiac events increases when transitioning from childhood to adulthood [27, 28]. Although symptomatology could not be compared due to lack of symptomatic patients in the current study, the risk of misdiagnosis would also include factors such as the total number of patients with LQTS, especially index cases, in a relevant age group. The current study included patients diagnosed with LQTS over a 15‐year period in a relatively large region in southern Sweden, including an academic paediatric cardiology centre. The age limit in the current study was necessary for the comparison with BHS patients. Under these circumstances, the few LQTS index cases, who were all asymptomatic, suggest that few LQTS patients exhibit symptoms within the age range where differential diagnosis between BHS and LQTS is relevant.

We identified two cases of syncope or seizures in conjunction with the suspicion of LQTS. Both patients were of a reasonable age for BHS, though the patient diagnosed with epilepsy was slightly older than the typical age of onset of BHS. Both patients were diagnosed with LQT1. Previous studies suggested that LQT2 is more likely to be misdiagnosed as a seizure disorder [20]. In the case of the patient with epilepsy, the suspicion of seizures contributed to a diagnostic delay of 16 months. The patient with BHS experienced no diagnostic delay, probably due to the known LQTS heredity. Increased diagnostic delays have been reported when LQTS is misdiagnosed as epilepsy [20], but LQTS symptoms were not misinterpreted as epilepsy in the current case. The LQTS diagnosis was suspected due to seizures but delayed because of the lack of arrhythmia on telemetry monitoring, despite borderline to prolonged QTc intervals.

### 4.1 Strengths and Limitations

A strength of this study is that the LQTS patient cohort was assembled using a population‐based approach. Furthermore, the LQTS cohort was collected within the same geographic region and during the same time period as the cohort with BHS, which served as the basis for the BHS management algorithm [22]. This alignment allowed for meaningful comparison between the LQTS and BHS cohorts. This study has some limitations. By using the same population as in our BHS study, we can now assert that no LQTS patients were misdiagnosed with BHS during the relevant time period. However, cases without an identified genetic variant, which is expected to be ~25% [31] in a population but was 0% in our study, or cases with diagnostic difficulties might have been missed for inclusion in this study. The objective was to investigate the symptoms and diagnostic pathways of patients with LQTS to identify factors similar to those in patients with BHS. These overlapping factors would warrant consideration in BHS management to reduce the risk of misdiagnosis. We did not retrospectively assess the accuracy of the LQTS diagnosis or ECG interpretations, focusing instead on the diagnostic pathway as experienced by patients. However, all patients were evaluated at least once by a pediatric cardiologist and underwent genetic testing.

The retrospective study design has the limitation of relying on nonstructured clinician data capture and parental reports. In addition, the sample size was limited by the number of cases available during the predetermined time period and geographic area. Existing LQTS registers will make it possible to increase the number of eligible patients with LQTS in the relevant age group for future studies. Studies on the effect of the guidelines on the usage of ECGs in BHS management are also needed.

Lastly, the inclusion period from 2004 to 2018 encompassed a period of significant advancement in genetic testing for LQTS. The management of LQTS patients today may differ from that in 2004. However, 12 of our patients had suspicion raised in 2010 or more recently.

## 5 Conclusion

None of the patients diagnosed with LQTS in the Skåne Region during the relevant period experienced diagnostic delays for LQTS due to suspicion or misdiagnosis of BHS. The results of this study do not contradict our previous conclusion that the risk of misdiagnosing LQTS as BHS is low if the suggested safeguards are included in the management of suspected BHS. However, the results indicate that a lower age limit for ECG, such as before 3 months old, may be considered as an addition to our BHS guidelines.

## Author Contributions

**Sanna Hellström Schmidt:** conceptualization, investigation, writing – original draft, methodology, writing – review and editing, project administration, formal analysis, visualization. **Ida Jeremiasen:** conceptualization, methodology, writing – review and editing, formal analysis. **Erik A. Eklund:** conceptualization, methodology, writing – review and editing, formal analysis. **Cornelis Jan Pronk:** conceptualization, investigation, writing – original draft, writing – review and editing, supervision.

## Funding

The Knut and Alice Wallenberg Foundation, the Medical Faculty at Lund University, and the Skåne Region are acknowledged for their generous financial support of C.J.P.

## Conflicts of Interest

The authors declare no conflicts of interest.

## Supporting information


**Table S1:** REDCap data entry form with field labels (variables) and choices (answer options) specified.

## Data Availability

The datasets generated during the current study are not publicly available due to the risk of identifying the included children, but they can be made available, when possible from an ethical and legal perspective, from the corresponding author on reasonable request.
