# Supplementary material for: Population‐Based Study Found Low Risk of Misdiagnosing Long QT Syndrome as Breath‐Holding Spells in Swedish Children
Source: Acta Paediatr. 2026 Jan 22;115(5):1116–25. doi: 10.1111/apa.70460 (PMC13063360; doi:10.1111/apa.70460)
Supplement: Supplementary file 1 — Table S1: REDCap data entry form with field labels (variables) and choices (answer options) specified. [file APA-115-1116-s001.docx]

Table S1 REDCap data entry form with field labels (variables) and choices (answer options) specified

| **REDCap field label/Variable name** | **REDCap choices/Answer options** |
| --- | --- |
| Record ID | Number (chronological order) |
| Sex | Multiple choice: Female / Male |
| Age at onset of symptoms (if symptomatic before diagnosis) | Number (months) |
| Suspicion of LQTS raised | Date |
| Answer on genetic testing | Date |
| Age at diagnosis | Number (months) |
| Diagnose date | Date |
| Comment on dates above | Text |
| Diagnosed where | Multiple choise: Emergency department / Cardiology department / Primary care (and then referred to cardiologist or pediatric clinic) / Private specialized care / Pediatric clinic (public care) / BVC (eng. Family center) / Other / Unknown |
| Other diagnosis before LQTS-diagnosis (wrongly diagnosed) | Multiple choice: Yes / No / Unknown |
| Other diagnoses, specified | Text |
| Medical history consistent with BHS or other seizure disorder besides LQTS? | Multiple choice: Yes / No / Unknown |
| Cause of discovery | Multiple choice: Screening because of heredity / Symptomatic / ECG taken for another reason / Other / Unknown |
| Medications at diagnosis | Multiple choice: Yes / No / Unknown |
| Medications at diagnosis, list | Text |
| Heredity for LQTS | Multiple choice: Yes / No / Unknown |
| Heredity for LQTS, specified | Checkboxes: First-degree relative / Second-degree relative / More distant relative / Unknown |
| Specification heredity | Checkboxes: Parent / Sibling (full sibling) / Child (of patient) / Grandparent / Parents’ sibling (aunt etc) / Sibling (half sibling) / Niece/nephew / Grandchild / Other |
| Heredity for LQTS, comment | Text |
| Heredity for BHS | Multiple choice: Yes / No / Unknown |
| Heredity for other seizure disorders | Multiple choice: Yes / No / Unknown |
| Symptomatic | Multiple choice: Yes / No / Unknown |
| Symptoms (if symptomatic) | Checkboxes: Dizziness / Loss of consciousness without seizure activity / Loss of consciousness with seizure activity / Cardiac arrest / Palpitations / Presyncope / Blurry vision / Other / Unknown |
| Symptomatic (if symptomatic), when | Checkboxes: Before diagnostic event / At diagnostic event / After diagnostic event / Unknown |
| Symptoms related to LQTS, BHS or other seizure disorder? | Multiple choice: Yes / No / Unknown |
| Symptomatic, comment | Text |
| Prodrome | Multiple choice: Yes / No / Unknown |
| Prodrome | Checkboxes: Before diagnostic event / At diagnostic event / After diagnostic event / Unknown |
| Loss of consciousness | Multiple choice: Yes / No / Unknown |
| Loss of consciousness | Checkboxes: Before diagnostic event / At diagnostic event / After diagnostic event / Unknown |
| Loss of consciousness, length (if not known exactly) | Checkboxes: <1 min / 1-5 min / >5 min / >10 min / Unknown |
| Loss of consciousness, length (minutes) | Number |
| Seizure activity | Multiple choice: Yes / No / Unknown |
| Seizure activity | Checkboxes: Before diagnostic event / At diagnostic event / After diagnostic event / Unknown |
| Seizure activity, length (if not known exactly) | Checkboxes: <1 min / 1-5 min / >5 min / >10 min / Unknown |
| Seizure activity, length (minutes) | Number |
| Seizure activity | Multiple choice: General / Focal / Unknown |
| Seizure activity | Checkboxes: Before diagnostic event / At diagnostic event / After diagnostic event / Unknown |
| Seizure activity, semiology | Checkboxes: Tonic / Clonic / Tonic-clonic / Myoclonic / Limp / Unknown |
| Postictal-like period | Multiple choice: Yes / No / Unknown |
| Postictal-like period | Checkboxes: Before diagnostic event / At diagnostic event / After diagnostic event / Unknown |
| Postictal-like period, length (if not known exactly) | Checkboxes: <1 min / 1-5 min / 6-30 min / 31-60 min / >60 min / Unknown |
| Postictal-like period, length (minutes) | Number |
| QT at diagnosis | Number |
| QT at diagnosis, interpretation by | Multiple choice: Machine / Physician / Unknown / Other |
| QTc at diagnosis | Number |
| QTc at diagnosis, interpretation by | Multiple choice: Machine / Physician / Unknown / Other |
| Date of ECG that QT and QTc above are based on | Date |
| ECG interpretation at diagnosis if noted in medical files (in body of text) | Text |
| QT and QTc times, some representative values | Numbers |
| QTc time, physicians’ assessment | Checkboxes: Normal / Long / Borderline value / Mostly normal but occasional long values / Mostly borderline but occasional long values / Other |
| Other ECG findings | Multiple choice: Yes / No / Unknown |
| Other ECG findings, comment | Text |
| ECGs interpreted by | Checkboxes: Machine only / Pediatrician / Pediatric cardiologist / Other / Unknown |
| ECG taken before diagnosis | Multiple choice: Yes / No / Unknown |
| ECG taken before diagnosis because of | Multiple choice: LQTS investigation / Other medical reason / Unknown |
| ECG before diagnosis (if taken) | Multiple choice: Normal / Pathologic / Unknown |
| ECG before diagnosis (if taken), comment | Text |
| Other investigations (to make the diagnosis) | Checkboxes: Blood samples / Radiological investigations / EEG / Cardiac investigations / Genetic test / Other / None / Unknown |
| Other investigations, comment | Test |
| Co-morbidity | Multiple choice: Yes / No / Unknown |
| Co-morbidity, disease group(s) | Checkboxes: Asthma and/or allergy / Gastro-intestinal disease / Renal disease / Hematological disease / Oncological disease / Other cardiac disease / Neurological disease / Rheumatic disease / Metabolic disease / Born prematurely / Endocrine disease / Immunological disease / Chronic infection / Skin disease / Other / Unknown |
| Co-morbidity, specified | Text |
| Screening for LQTS among relatives | Checkboxes: Before patient’s diagnosis / At the same time as the patient’s diagnosis / After patient’s diagnosis |
| If screening for LQTS among relatives conducted after patient diagnosed with LQTS, was it because of diagnosis found in the patient? | Multiple choice: Yes / No / Unknown |
| Relative with LQTS found because of screening after patient was diagnosed with LQTS? | Multiple choice: Yes / No / Unknown |
| Treatment | Multiple choice: Yes / No / Unknown |
| Treatment, modality | Checkboxes: Medications for example betablockers / Implantable cardioverter defibrillator / Other / Unknown |
| Treatment, specified | Text |
| Follow up | Multiple choice: Yes / No / Unknown |
| Date of last checkup (concerning LQTS) | Date |
| Follow up length (diagnosis to last checkup) | Number (months) |
| Patient deceased? | Multiple choice: Yes / No / Unknown |
| Comment | Text |
| Date information was gathered in medical files | Date |
